# Venous resection increases risk of chyle leak after total pancreatectomy for pancreatic tumors

**DOI:** 10.1186/s12957-024-03451-0

**Published:** 2024-06-28

**Authors:** Tianyu Li, Chen Lin, Bangbo Zhao, Zeru Li, Yutong Zhao, Xianlin Han, Menghua Dai, Junchao Guo, Weibin Wang

**Affiliations:** Department of General Surgery, State Key Laboratory of Complex Severe and Rare Diseases, Peking Union Medical College Hospital, Chinese Academy of Medical Sciences and Peking Union Medical College, Beijing, China

## Abstract

**Background:**

Existing research on chyle leak (CL) after pancreatic surgery is mostly focused on pancreaticoduodenectomy and lacks investigation on total pancreatectomy (TP). This study aimed to explore potential risk factors of CL and develop a predictive model for patients with pancreatic tumor undergoing TP.

**Methods:**

This retrospective study enrolled 90 consecutive patients undergoing TP from January 2015 to December 2023 at Peking Union Medical College Hospital. According to the inclusion criteria, 79 patients were finally included in the following analysis. The LASSO regression and multivariate logistic regression analysis were performed to identify risk factors associated with CL and construct a predictive nomogram. Then, the ROC analysis, calibration curve, decision curve analysis (DCA), and clinical impact curve (CIC) were performed to assess its discrimination, accuracy, and efficacy. Due to the small sample size, we adopted the bootstrap resampling method with 500 repetitions for validation. Lastly, we plotted and analyzed the trend of postoperative drainage volume in CL patients.

**Results:**

We revealed that venous resection (OR = 4.352, 95%CI 1.404–14.04, *P* = 0.011) was an independent risk factor for CL after TP. Prolonged operation time (OR = 1.473, 95%CI 1.015–2.237, *P* = 0.052) was also associated with an increased incidence of CL. We included these two factors in our prediction model. The area under the curve (AUC) was 0.752 (95%CI 0.622–0.874) after bootstrap. The calibration curve, DCA and CIC showed great accuracy and clinical benefit of our nomogram. In patients with CL, the mean drainage volume was significantly higher in venous resection group and grade B CL group.

**Conclusion:**

Venous resection was an independent risk factor for chyle leak after TP. Patients undergoing vascular resection during TP should be alert for the occurrence of CL after surgery. We then constructed a nomogram consisted of venous resection and operation time to predict the odds of CL in patients undergoing TP.

**Supplementary Information:**

The online version contains supplementary material available at 10.1186/s12957-024-03451-0.

## Introduction

Fat is digested and absorbed from the small intestine into the lymph, and the fat particles with a milky appearance, called chyle, are often collected in the chylous vessels and blood [1]. Chyle leak is a common complication following abdominal surgery, resulting from the disruption of abdominal lymphatics [2]. Pancreatic surgery often results in chyle leakage due to damage to the cisterna chyli or surrounding lymphatics situated around the pancreatic head and neck [3], which prolonged the patient's hospital stay and increasing financial burden [4]. Chyle leak is defined as milky-appearing drainage fluid after postoperative day 3 (POD 3) with a triglyceride content of at least 1.2 mmol/L (110 mg/dL) by the International Study Group of Pancreatic Surgery (ISGPS) in 2017 [5]. A nationwide analysis in Dutch included 2159 patients undergoing pancreatoduodenectomy and reported 7.0% CL rate. They also found that vascular resection and open surgery were independent risk predictors for CL [2]. Another large cohort study included 3324 patients after pancreatic surgery and identified diabetes, malignancy, distal pancreatectomy, operation time, and pancreatic fistula as independent risk factors for chyle leak [3]. However, due to the difference in surgical resection extent and difficulty, predictors of CL after total pancreatectomy (TP) are likely to be different from other pancreatectomies. Current study lacks targeted exploration for CL after TP. Therefore, the aim of this research was to discover the risk factors and construct a prediction model for CL after TP.

## Materials and methods

### Study population

**Fig. 1:**
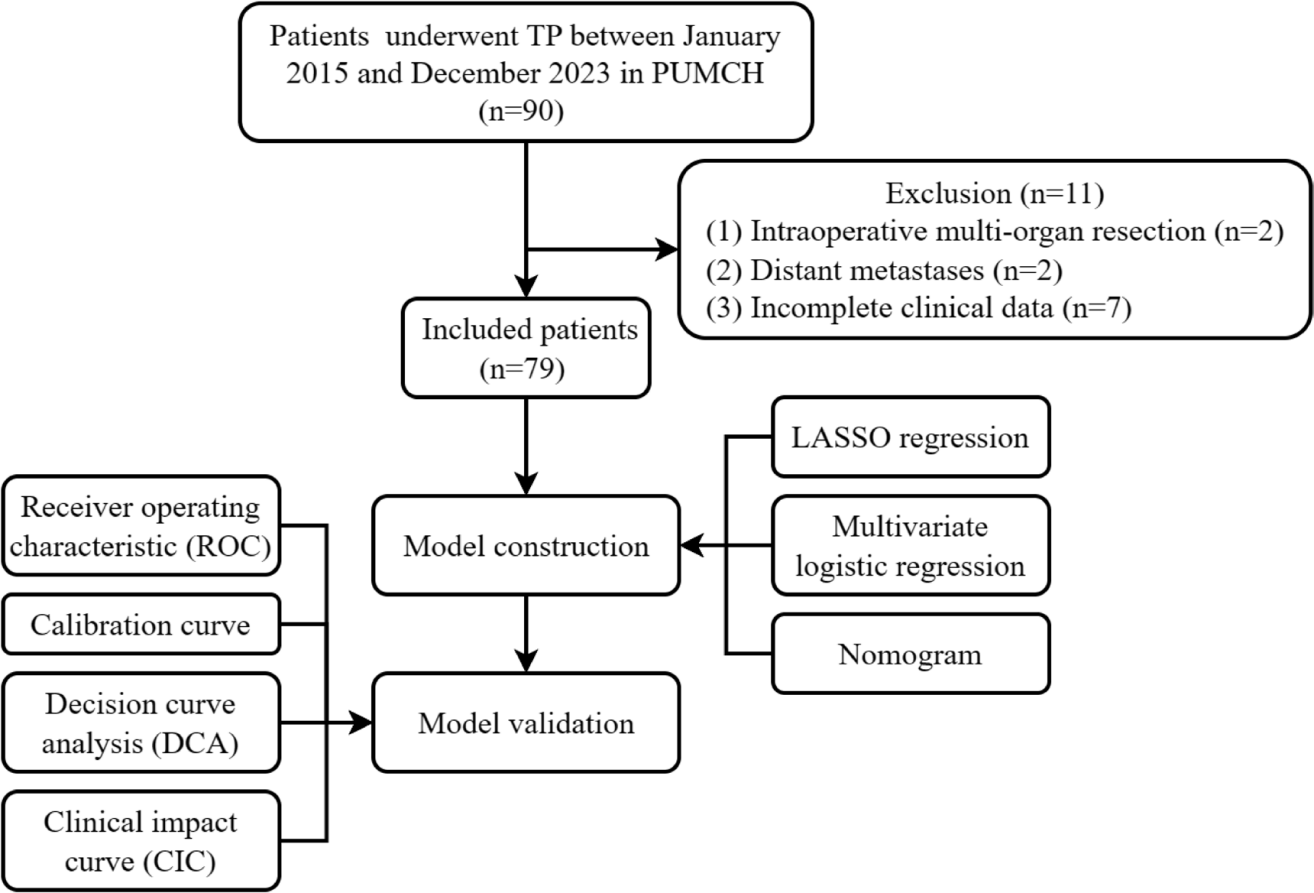
Flowchart of the model building and validation process

Ninety consecutive patients underwent total pancreatectomy (TP) in Peking Union Medical College Hospital between January 2015 and December 2023 were retrospectively collected in our research. The flowchart of the study was shown in Fig. 1. The inclusion and exclusion criteria were as outlined below. Inclusion criteria: Patients with pancreatic tumor undergoing TP. Exclusion criterion: (1) patients undergoing multi-organ resection during TP; (2) patients with distant metastases; (3) patients with incomplete clinical information. A total of 79 patients were finally included in this study. There was no change to the surgical method during the study period.

### Clinical data collection and definition

Patients information were collected through the medical record system of our hospital, including age, gender, BMI (Body Mass Index), ASA (American Society of Anesthesiologists) classification, past medical history (hypertension, diabetes, chronic heart disease, and pancreatitis), history of smoking and drinking, history of preoperative treatment (neoadjuvant therapy and biliary drainage), intraoperative and surgical details (operation time, bleeding amount, blood transfusion amount, fluid replacementamount, minimally invasive, and venous resection (TP with venous resection was defined according to the Heidelberg type 2 categorization [6])), and pathology information (harvested and positive lymph nodes, resection margin, and nature of the lesions). Chyle leak was defined as abdominal drainage output of milky-colored fluid after postoperative day 3 with a triglyceride content ≥ 1.2 mmol/L or chylomicron qualitative test positive. Based on the required management, three different severity ratings were established [5]. Grade A was defined as no intervention needed apart from daily oral dietary restriction; grade B, prolonged hospital stay, restrictive enteral nutrition, total parenteral nutrition, somatostatin and its analogues (octreotide), maintenance or placement of percutaneous drains; and grade C, patient needed further invasive treatment, intensive care unit admission, or died because of chyle leakage.

### Statistical method

We adopted the “glmnet” R package for LASSO regression analysis and the “glm” R package was for multiple logistic regression to identify the independent risk factors associated with chyle leak in patients undergoing TP. A nomogram was established using the “rms” R package to represent the risk of chyle leak in these patients. The model was validated by 500 repetitions bootstrap resampling methods. The “pROC” and “rms” R package was used to plot ROC and calibration curve. The “rmda” R package was used to perform DCA and CIC analyses. Statistical analysis was performed by R software package (version 4.2.1). Fisher’s exact test and chi-square test was used for categorical variables. T test and Mann–Whitney U test was used for continuous variables. A two-sided *p*-value < 0.05 was considered statistically significant.

## Results

### Patient characteristics

A total of 79 patients were enrolled in this study after exclusion, of which 20 patients developed chyle leak (CL). Among them, 10 patients were grade A, 10 patients were grade B, and no patients had grade C CL. The median and interquartile range of abdominal drain fluid TG was 2.86 [1.86; 4.99] mmol/L in CL group. Table 1 showed the perioperative variables in the CL and non-CL cohorts, with significant differences observed in operation time, and venous resection (*p* < 0.05). The number of lymph node harvested was also higher in CL patients (*p* = 0.069). Table 2 demonstrated the surgical outcome for patients undergoing TP. The hospital stay was prolonged in the CL group compared to the non-CL group, although no statistical difference was observed (*p* = 0.08). Also, there was no statistically significant difference in postoperative complication morbidity and mortality.

**Table 1:** Perioperative characteristics of TP patients in the Non-CL and CL cohorts

Variables	Total cohort (*N* = 79)	Non-CL cohort (*N* = 59)	CL cohort (*N* = 20)	*P*
Gender				0.847
Female	40 (50.63%)	29 (49.15%)	11 (55.00%)	
Male	39 (49.37%)	30 (50.85%)	9 (45.00%)	
Age, year	63.00 [54.50;68.50]	63.00 [55.00;69.50]	61.00 [51.75;67.00]	0.531
ASA Classification				0.136
1	3 (3.85%)	2 (3.45%)	1 (5.00%)	
2	58 (74.36%)	46 (79.31%)	12 (60.00%)	
3	17 (21.79%)	10 (17.24%)	7 (35.00%)	
BMI, kg/m^2^	22.30 (2.97)	22.31 (2.79)	22.29 (3.53)	0.982
Hypertension				1.000
No	44 (55.70%)	33 (55.93%)	11 (55.00%)	
Yes	35 (44.30%)	26 (44.07%)	9 (45.00%)	
Diabetes				1.000
No	42 (53.16%)	31 (52.54%)	11 (55.00%)	
Yes	37 (46.84%)	28 (47.46%)	9 (45.00%)	
Chronic heart disease				1.000
No	70 (88.61%)	52 (88.14%)	18 (90.00%)	
Yes	9 (11.39%)	7 (11.86%)	2 (10.00%)	
Pancreatitis				0.112
No	69 (87.34%)	54 (91.53%)	15 (75.00%)	
Yes	10 (12.66%)	5 (8.47%)	5 (25.00%)	
Neoadjuvant therapy				1.000
No	75 (94.94%)	56 (94.92%)	19 (95.00%)	
Yes	4 (5.06%)	3 (5.08%)	1 (5.00%)	
Smoker				0.854
No	48 (60.76%)	35 (59.32%)	13 (65.00%)	
Yes	31 (39.24%)	24 (40.68%)	7 (35.00%)	
Drinker				1.000
No	55 (69.62%)	41 (69.49%)	14 (70.00%)	
Yes	24 (30.38%)	18 (30.51%)	6 (30.00%)	
Obstructive jaundice				0.914
No	58 (73.42%)	44 (74.58%)	14 (70.00%)	
Yes	21 (26.58%)	15 (25.42%)	6 (30.00%)	
Preoperative biliary drainage				0.456
No	68 (86.08%)	52 (88.14%)	16 (80.00%)	
Yes	11 (13.92%)	7 (11.86%)	4 (20.00%)	
Operation time, hour	6.68 (1.47)	6.47 (1.50)	7.30 (1.23)	0.018
Intraoperative bleeding, ml	500.00 [300.00;800.00]	400.00 [300.00;800.00]	650.00 [400.00;800.00]	0.286
Intraoperative blood transfusion, ml	400.00 [0.00;700.00]	400.00 [0.00;800.00]	100.00 [0.00;400.00]	0.686
Intraoperative fluid replacement, ml	3950.00 [3350.00;4700.00]	3900.00 [3100.00;4400.00]	4500.00 [3875.00;5200.00]	0.026
Minimally invasive				0.914
No	58 (73.42%)	44 (74.58%)	14 (70.00%)	
Yes	21 (26.58%)	15 (25.42%)	6 (30.00%)	
Venous resection				0.014
No	58 (73.42%)	48 (81.36%)	10 (50.00%)	
Yes	21 (26.58%)	11 (18.64%)	10 (50.00%)	
Positive lymph nodes	0.00 [0.00;2.00]	0.00 [0.00;2.00]	1.00 [0.00;4.00]	0.175
Harvested lymph nodes	25.00 [16.00;34.50]	23.00 [14.50;32.50]	27.00 [23.00;40.00]	0.069
Positive lymph node ratio	0.00 [0.00;0.09]	0.00 [0.00;0.06]	0.03 [0.00;0.10]	0.269
Resection margin				0.729
No	66 (83.54%)	50 (84.75%)	16 (80.00%)	
Yes	13 (16.46%)	9 (15.25%)	4 (20.00%)	
Malignancy:				0.131
No	19 (24.05%)	17 (28.81%)	2 (10.00%)	
Yes	60 (75.95%)	42 (71.19%)	18 (90.00%)

**Table 2:** Surgical outcome of TP patients in the Non-CL and CL cohorts

Variables	Total cohort (*N* = 79)	Non-CL cohort (*N* = 59)	CL cohort (*N* = 20)	*P*
Length of hospital stay	19.00 [17.00;25.00]	19.00 [15.50;23.50]	21.00 [19.00;26.25]	0.086
Length of ICU stay	1.00 [0.00;1.50]	1.00 [0.00;2.00]	1.00 [0.00;1.00]	0.844
Clavien-Dindo ≥ 3a				0.435
No	70 (88.61%)	51 (86.44%)	19 (95.00%)	
Yes	9 (11.39%)	8 (13.56%)	1 (5.00%)	
Delayed gastric emptying				0.186
No	56 (70.89%)	39 (66.10%)	17 (85.00%)	
Yes	23 (29.11%)	20 (33.90%)	3 (15.00%)	
Pancreatic fistula				-
No	79 (100.00%)	59 (100.00%)	20 (100.00%)	
Postpancreatectomy hemorrhage				1.000
No	77 (97.47%)	57 (96.61%)	20 (100.00%)	
Yes	2 (2.53%)	2 (3.39%)	0 (0.00%)	
Intra-abdominal infection				0.729
No	66 (83.54%)	50 (84.75%)	16 (80.00%)	
Yes	13 (16.46%)	9 (15.25%)	4 (20.00%)	
Incision infection				1.000
No	75 (94.94%)	56 (94.92%)	19 (95.00%)	
Yes	4 (5.06%)	3 (5.08%)	1 (5.00%)	
Pleural effusion / Lung infection				0.729
No	66 (83.54%)	50 (84.75%)	16 (80.00%)	
Yes	13 (16.46%)	9 (15.25%)	4 (20.00%)	
Urinary tract infection				0.445
No	77 (97.47%)	58 (98.31%)	19 (95.00%)	
Yes	2 (2.53%)	1 (1.69%)	1 (5.00%)	
30-day mortality				1.000
No	78 (98.73%)	58 (98.31%)	20 (100.00%)	
Yes	1 (1.27%)	1 (1.69%)	0 (0.00%)	

### Identification of independent risk factor for predicting CL

**Fig. 2:**
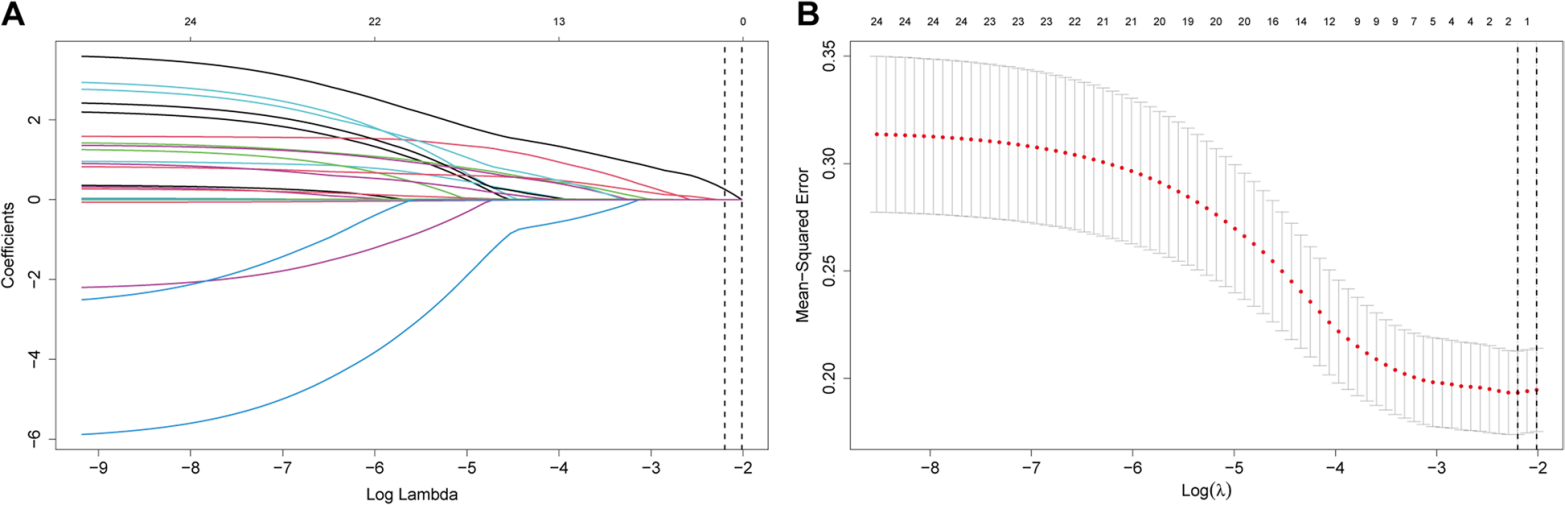
Variables selection by the LASSO regression. **A** LASSO coefficient profiles of the clinical features. **B** The optimal penalization coefficient lambda was generated via tenfold cross-validation

Of the 24 variables, 2 predictive features were selected using the LASSO regression analysis (Fig. 2A and B). The Lambda.1se identified a model with optimal predictive ability with minimal predictors, including operation time and venous resection. These two factors screened from LASSO were then applied to identify independent risk factors by multivariable logistic regression analysis, which venous resection (OR = 4.352, 95% CI 1.404–14.04, *P* = 0.011) was identified as statistically significant risk factor for CL, as demonstrated in Table 3.

**Table 3:** Multivariate logistic regression

Characteristics	Estimate	SE	OR	95% CI	*P*-value
(Intercept)	-4.215	1.44553	0.015	0.014 (0.000–0.205)	0.004
Operation time, hour	0.387	0.19873	1.473	1.472 (1.015–2.237)	0.052
Venous resection	1.471	0.58129	4.352	4.351 (1.404–14.04)	0.011

*SE* standard error, *OR* odds ratio

### Predictive model construction

**Fig. 3:**
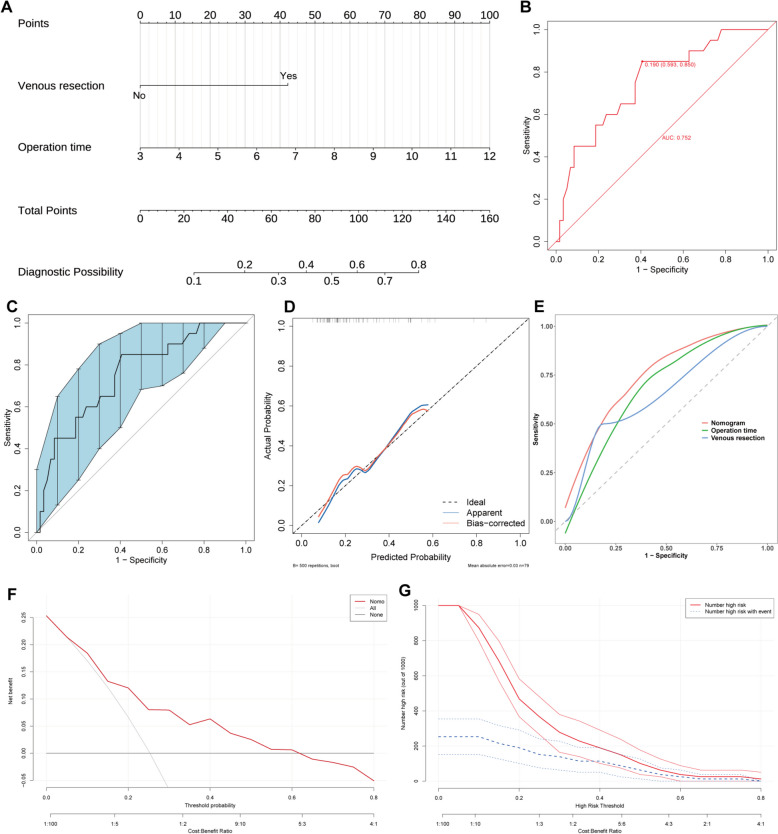
Nomogram construction and validation. **A** Nomogram for predicting CL after TP. **B** ROC curve for the prediction model. **C** ROC curve validation by 500 times bootstrap replicates. **D** Calibration curves of the risk nomogram. **E** Rationality curve analysis for the CL risk nomogram. **F** Decision curve analysis. ‘All’ refers to that all patients have CL and ‘none’ means no patient has CL. **G** The CIC curve of the nomogram. The red solid line refers to the total patients regarded as high risk for each risk threshold. The blue dashed line refers to those would be actual CL patients

Although there is no statistical difference for operation time (OR = 1.473, 95% CI 1.015–2.237, *P* = 0.052), we still believe that it is associated with CL occurrence and included these two risk factors in a nomogram (Fig. 3A), aiming to predict the risk of CL for patients who underwent TP. Figure 3B showed our model. Because of the small sample size, we did not perform random assignment to training and validation sets. The area under the curve (AUC) was 0.752 (95% CI 0.593–0.850) (Fig. 3B). After 500 times bootstrap replicates, the observed AUC was 0.752 (95% CI 0.621–0.859) (Fig. 3C), indicating great discriminative ability. We also compared the AUC value of the nomogram with other single predictors, as depicted in Fig. 3D. Note that the AUC of the individual predictors was consistently smaller than the predictive model, underscoring the robust performance of this model. The calibration curves are shown in Fig. 3E, which showed an ideal consistency between the prediction and actual observation after bootstrap replicates. The Hosmer–Lemeshow goodness-of-fit (GOF) test showed X-squared values of 3.3216 (*p* = 0.9126), suggesting a good fit in our model. The clinical efficacy of our nomogram was evaluated by DCA and CIC curve with fivefold cross-validation and 500 times bootstrap (Figs. 3F and G). The decision curve analysis demonstrated high net benefits in our cohort. Within the probabilities ranging from 5 to 62%, the model outperforms the “treat-all” or “treat-non” strategies, indicating increased net benefits.

### Venous resection also increases risk of Grade B CL after TP

Since grade B CL is more important in the clinical practice, we compared the perioperative characteristics and surgical outcome for TP patients in the Non-CL/Grade A CL and Grade B CL cohorts. The results showed that the proportion of venous resection (*P* = 0.019), the number of harvested lymph nodes (*P* = 0.032), and intraoperative fluid replacement (*P* = 0.020) were higher in the grade B CL group (Supplement Table 1). Besides, there was no significant difference in surgical outcomes between the two groups (Supplement Table 2). After backward multivariate logistic regression analysis, venous resection (OR = 4.118, 95% CI 0.971–18.80, *P* = 0.055) and harvested lymph nodes (OR = 1.049, 95% CI 1.000–1.104, *P* = 0.051) were selected as risk factors for Grade B CL (Supplement Table 3). However, since only 10 patients developed grade B CL in our research, it was insufficient to further construct a valid predictive model.

### Postoperative drainage volume analysis in CL patients

**Fig. 4:**
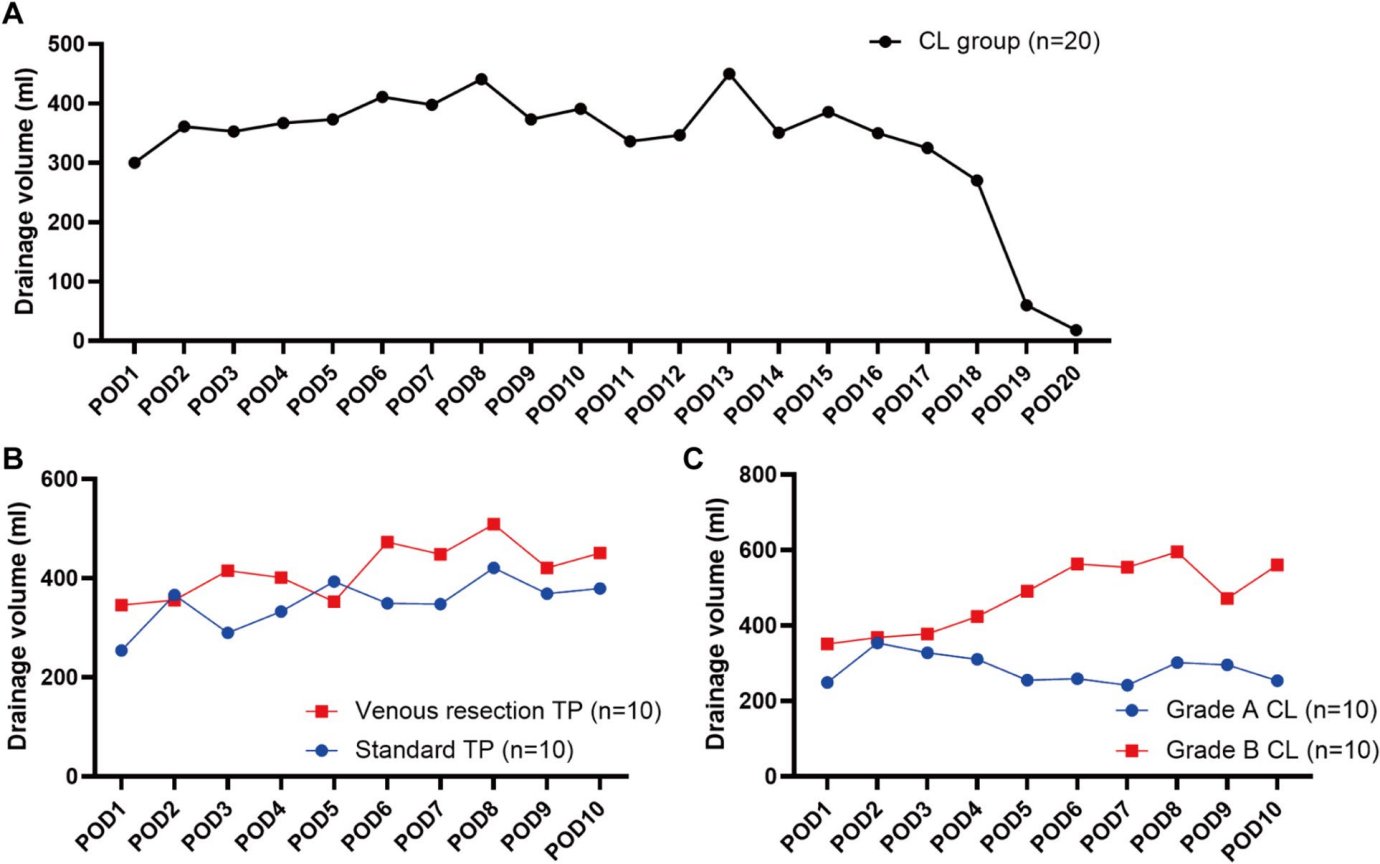
**A** The changing trend of mean drainage volume in CL patients from POD1 to POD20. **B** The mean drainage volume for patients after standard TP and venous resection TP from POD1 to POD10. **C** The mean drainage volume of grade A and B CL from POD1 to POD10

Numerous research have shown the link between CL and oral diet, and the characteristics of drainage fluid change with patient's food intake [7–10]. In addition to the color of the drainage fluid, the volume is also an important indicator of CL. However, no study has reported the volume of drainage fluid in patients with CL after TP. First, we calculated the mean drainage volume of CL patients within 20 days after surgery (Fig. 4A). We found that the mean drainage volume in the CL group remained above 300 ml for a long time after surgery, but seemed to start decreasing after POD14. Then, since venous resection was the independent risk factor for CL in our model, we discovered that the mean drainage volume in venous resection TP group was generally higher than standard TP group (Fig. 4B). Also, grade B CL group also had significantly higher mean drainage volume than grade A CL (Fig. 4C).

## Discussion

This retrospective study demonstrated a 25.3% incidence of grade A/B chyle leak (CL), and no grade C CL was observed. Venous resection was identified as risk factor for CL after total pancreatectomy (TP) in the present study. A single-center retrospective study enrolled 2159 patients underwent PD and aimed to assess the risk factors of chyle leak [2]. Since only a small number of arterial resections (*n* = 27) were performed in this research, only venous resection was recognized as independent risk factor (*P* < 0.001). In patients with CL, venous resection rate was 25.9% and significantly higher than non-CL group (13.6%). Another lager cohort study also identified vascular resection as risk predictor for CL after pancreatic surgery [11]. The elevated risk of CL may be partially attributed to the skeletonization of nearby vascular structures and extensive retroperitoneal dissection necessary during PV/SMV reconstruction, which could cause damage to cisterna chyli and peripheral lymphatic vessels. Also, venous reconstruction may lead to a higher likelihood of local thrombosis. Several studies have reported that postoperative PV/SMV thrombosis was significantly correlated with a higher incidence of CL [12, 13].

Our study also revealed that prolonged operation time was linked with higher CL rate (*P* = 0.052). Similarly, Assumpção et al. [11], Tabchouri et al. [8], Strobel et al. [3], Paiella et al. [14], and Russell et al. [15] reported the same result in their univariate or multivariate analyses. Except for the skill and technique of the surgeon, operation time is influenced by surrounding inflammation in the operative area, which may negatively affect lymphatic inflow and separating adhesions may damage lymphatic vessels [16], resulting CL after surgery. Also, operative time is affected by many variables including tumor stage, BMI, and surgical detail (extensive lymph nodes dissection, multi-organ resection or vascular reconstruction) etc.

The number of harvested lymph nodes were also found as risk factors for grade B CL, which was in accordance with previous literature [17–19]. Due to the extensive range of surgical resection, especially lymph node resection, this could directly lead to injury of cisterna chyli and lymphatic vessels [17], which increased incidence of CL. Patients underwent aortocaval lymph node sampling showed high CL rate after surgery [8, 20, 21]. Unfortunately, the absence of related pathology data hindered us from considering this aspect. Furthermore, a nonsignificant incidence of CL was observed after minimally invasive TP as compared with open surgery.

Post-operative abdominal drainage volume was significantly higher in CL patients than non-CL patients [17, 22]. Shyr et al. enrolled 34 CL patients undergoing PD and discovered that drainage volume in the CL group remained constant with a median of 240 mL on both POD1 and POD7 [17]. However, no current research has focused on drainage volume after TP. In our analysis, we quantified the volume of abdominal drainage in patients with CL over a period of 20 days postoperatively, providing a preliminary reference for early detection of CL. Moreover, in CL group, postoperative drainage volume was significantly increased in patients who underwent venous resection, supporting our prediction of it as an independent risk factor for CL.

This is a pioneering study to investigate the risk factors for CL after TP and construct a predictive model. Besides, we provided specific data on postoperative drainage according to venous resection and CL grade. We believe these could serve as a preliminary exploration and lay foundation for subsequent high-quality studies with larger sample sizes. There are still several limitations in this study as the collected data is restricted to only our hospital with a small sample size and has not yet been externally validated by other centers. Additionally, our model is established using clinical data we can collect, and it is possible that there are superior clinical markers that might enhance its ability to predict CL. Also, patients classified as Grade C CL were not included in this research and thus we did not introduce the definition of clinically relevant CL (grade B/C) for risk stratification. More importantly, avoiding CL and advancing its treatment window are significant challenges that call for further investigation and high-level research.

## Conclusion

Venous resection was identified as independent risk factor for chyle leak after TP. We constructed a nomogram consisted of venous resection and operation time to predict the odds of CL in patients undergoing TP. We also performed further analyses for grade B CL only and identified venous resection and harvested lymph nodes as risk factors for grade B CL. Besides, in venous resection TP group, the mean drainage volume was higher than standard TP group. Similarly, grade B CL group had significantly higher mean drainage volume than grade A CL.

### Supplementary Information

Supplementary Material 1.

## Data Availability

No datasets were generated or analysed during the current study.

## Abbreviations

CL: Chyle leak
TP: Total pancreatectomy
PD: Pancreaticoduodenectomy
POD: Postoperative day
LASSO: Least absolute shrinkage and selection operator
ROC: Receiver operating characteristic curve
DCA: Decision curve analysis
CIC: Clinical impact curve
