# Supplementary material for: Venous resection increases risk of chyle leak after total pancreatectomy for pancreatic tumors
Source: World J Surg Oncol. 2024 Jun 28;22:174. doi: 10.1186/s12957-024-03451-0 (PMC11214213; doi:10.1186/s12957-024-03451-0)
Supplement: Supplementary file 1 — Supplementary Material 1. [file 12957_2024_3451_MOESM1_ESM.docx]

Supplement Table 1: Perioperative characteristics of TP patients in the Non-CL/Grade A CL and Grade B CL cohorts.

| Variables | Total cohort  (N=79) | Non-CL/Grade A CL cohort (N=69) | Grade B CL cohort  (N=10) | P |
| --- | --- | --- | --- | --- |
| Gender |  |  |  | 1.000 |
| Female | 40 (50.63%) | 35 (50.72%) | 5 (50.00%) |  |
| Male | 39 (49.37%) | 34 (49.28%) | 5 (50.00%) |  |
| Age, year | 63.00 [54.50;68.50] | 63.00 [56.00;69.00] | 53.50 [50.25;64.75] | 0.075 |
| ASA Classification |  |  |  | 0.128 |
| 1 | 3 (3.85%) | 2 (2.94%) | 1 (10.00%) |  |
| 2 | 58 (74.36%) | 53 (77.94%) | 5 (50.00%) |  |
| 3 | 17 (21.79%) | 13 (19.12%) | 4 (40.00%) |  |
| BMI, kg/m^2^ | 22.30 (2.97) | 22.26 (2.90) | 22.58 (3.61) | 0.794 |
| Hypertension |  |  |  | 1.000 |
| No | 44 (55.70%) | 38 (55.07%) | 6 (60.00%) |  |
| Yes | 35 (44.30%) | 31 (44.93%) | 4 (40.00%) |  |
| Diabetes |  |  |  | 1.000 |
| No | 42 (53.16%) | 37 (53.62%) | 5 (50.00%) |  |
| Yes | 37 (46.84%) | 32 (46.38%) | 5 (50.00%) |  |
| Chronic heart disease |  |  |  | 1.000 |
| No | 70 (88.61%) | 61 (88.41%) | 9 (90.00%) |  |
| Yes | 9 (11.39%) | 8 (11.59%) | 1 (10.00%) |  |
| Pancreatitis |  |  |  | 0.607 |
| No | 69 (87.34%) | 61 (88.41%) | 8 (80.00%) |  |
| Yes | 10 (12.66%) | 8 (11.59%) | 2 (20.00%) |  |
| Neoadjuvant therapy |  |  |  | 1.000 |
| No | 75 (94.94%) | 65 (94.20%) | 10 (100.00%) |  |
| Yes | 4 (5.06%) | 4 (5.80%) | 0 (0.00%) |  |
| Smoker |  |  |  | 0.502 |
| No | 48 (60.76%) | 43 (62.32%) | 5 (50.00%) |  |
| Yes | 31 (39.24%) | 26 (37.68%) | 5 (50.00%) |  |
| Drinker |  |  |  | 0.482 |
| No | 55 (69.62%) | 49 (71.01%) | 6 (60.00%) |  |
| Yes | 24 (30.38%) | 20 (28.99%) | 4 (40.00%) |  |
| Obstructive jaundice |  |  |  | 0.443 |
| No | 58 (73.42%) | 52 (75.36%) | 6 (60.00%) |  |
| Yes | 21 (26.58%) | 17 (24.64%) | 4 (40.00%) |  |
| Preoperative biliary drainage |  |  |  | 0.624 |
| No | 68 (86.08%) | 60 (86.96%) | 8 (80.00%) |  |
| Yes | 11 (13.92%) | 9 (13.04%) | 2 (20.00%) |  |
| Operation time, hour | 6.68 (1.47) | 6.57 (1.45) | 7.45 (1.48) | 0.103 |
| Intraoperative bleeding, ml | 500.00 [300.00;800.00] | 500.00 [300.00;800.00] | 500.00 [225.00;775.00] | 0.733 |
| Intraoperative blood transfusion, ml | 400.00 [0.00;700.00] | 400.00 [0.00;800.00] | 0.00 [0.00;300.00] | 0.287 |
| Intraoperative fluid replacement, ml | 3950.00 [3350.00;4700.00] | 3900.00 [3200.00;4400.00] | 4750.00 [4225.00;5350.00] | **0.020** |
| Minimally invasive |  |  |  | 1.000 |
| No | 58 (73.42%) | 50 (72.46%) | 8 (80.00%) |  |
| Yes | 21 (26.58%) | 19 (27.54%) | 2 (20.00%) |  |
| Venous resection |  |  |  | **0.019** |
| No | 58 (73.42%) | 54 (78.26%) | 4 (40.00%) |  |
| Yes | 21 (26.58%) | 15 (21.74%) | 6 (60.00%) |  |
| Positive lymph nodes | 0.00 [0.00;2.00] | 0.00 [0.00;2.00] | 1.00 [0.00;4.75] | 0.199 |
| Harvested lymph nodes | 25.00 [16.00;34.50] | 23.00 [16.00;32.00] | 36.50 [25.00;46.00] | **0.032** |
| Positive lymph node ratio | 0.00 [0.00;0.09] | 0.00 [0.00;0.06] | 0.06 [0.00;0.10] | 0.319 |
| Resection margin |  |  |  | 0.666 |
| No | 66 (83.54%) | 58 (84.06%) | 8 (80.00%) |  |
| Yes | 13 (16.46%) | 11 (15.94%) | 2 (20.00%) |  |
| Malignancy: |  |  |  | 0.437 |
| No | 19 (24.05%) | 18 (26.09%) | 1 (10.00%) |  |
| Yes | 60 (75.95%) | 51 (73.91%) | 9 (90.00%) |  |

Supplement Table2: Surgical outcome of TP patients in the Non-CL/Grade A CL and Grade B CL cohorts.

| Variables | Total cohort  (N=79) | Non-CL/Grade A CL cohort (N=69) | Grade B CL cohort  (N=10) | P |
| --- | --- | --- | --- | --- |
| Length of hospital stay | 19.00 [17.00;25.00] | 19.00 [17.00;23.00] | 23.00 [19.50;30.50] | 0.174 |
| Length of ICU stay | 1.00 [0.00;1.50] | 1.00 [0.00;2.00] | 1.00 [0.25;1.00] | 0.870 |
| Clavien-Dindo ≥3a |  |  |  | 1.000 |
| No | 70 (88.61%) | 61 (88.41%) | 9 (90.00%) |  |
| Yes | 9 (11.39%) | 8 (11.59%) | 1 (10.00%) |  |
| Delayed gastric emptying |  |  |  | 0.266 |
| No | 56 (70.89%) | 47 (68.12%) | 9 (90.00%) |  |
| Yes | 23 (29.11%) | 22 (31.88%) | 1 (10.00%) |  |
| Pancreatic fistula |  |  |  | - |
| No | 79 (100.00%) | 69 (100.00%) | 10 (100.00%) |  |
| Postpancreatectomy hemorrhage |  |  |  | 1.000 |
| No | 77 (97.47%) | 67 (97.10%) | 10 (100.00%) |  |
| Yes | 2 (2.53%) | 2 (2.90%) | 0 (0.00%) |  |
| Intra-abdominal infection |  |  |  | 1.000 |
| No | 66 (83.54%) | 57 (82.61%) | 9 (90.00%) |  |
| Yes | 13 (16.46%) | 12 (17.39%) | 1 (10.00%) |  |
| Incision infection |  |  |  | 0.425 |
| No | 75 (94.94%) | 66 (95.65%) | 9 (90.00%) |  |
| Yes | 4 (5.06%) | 3 (4.35%) | 1 (10.00%) |  |
| Pleural effusion / Lung infection |  |  |  | 0.355 |
| No | 66 (83.54%) | 59 (85.51%) | 7 (70.00%) |  |
| Yes | 13 (16.46%) | 10 (14.49%) | 3 (30.00%) |  |
| Urinary tract infection |  |  |  | 1.000 |
| No | 77 (97.47%) | 67 (97.10%) | 10 (100.00%) |  |
| Yes | 2 (2.53%) | 2 (2.90%) | 0 (0.00%) |  |
| 30-day mortality |  |  |  | 1.000 |
| No | 78 (98.73%) | 68 (98.55%) | 10 (100.00%) |  |
| Yes | 1 (1.27%) | 1 (1.45%) | 0 (0.00%) |  |

Supplement Table 3: Multivariate logistic regression

| Characteristics | Estimate | SE | OR | 95% CI | P-value |
| --- | --- | --- | --- | --- | --- |
| (Intercept) | -4.003 | 0.97584 | 0.018 | 0.018 (0.001-0.099) | 0 |
| Harvested lymph nodes | 0.048 | 0.02449 | 1.049 | 1.048 (1.000-1.104) | 0.051 |
| Venous resection | 1.415 | 0.73812 | 4.118 | 4.117 (0.971-18.80) | 0.055 |

SE standard error, OR odds ratio
